# Induction of male neogametogenesis in a three-dimensional microenvironment supporting successful fertilization and proper embryo development

**DOI:** 10.1093/molehr/gaag036

**Published:** 2026-06-04

**Authors:** Eros Lari, Lily Ng, Philip Xie, Stephanie Cheung, Sabrina Bains, Zev Rosenwaks, Gianpiero D Palermo

**Affiliations:** Ronald O. Perelman and Claudia Cohen Center for Reproductive Medicine, Weill Cornell Medicine, New York, NY, USA; Ronald O. Perelman and Claudia Cohen Center for Reproductive Medicine, Weill Cornell Medicine, New York, NY, USA; Ronald O. Perelman and Claudia Cohen Center for Reproductive Medicine, Weill Cornell Medicine, New York, NY, USA; Ronald O. Perelman and Claudia Cohen Center for Reproductive Medicine, Weill Cornell Medicine, New York, NY, USA; Ronald O. Perelman and Claudia Cohen Center for Reproductive Medicine, Weill Cornell Medicine, New York, NY, USA; Ronald O. Perelman and Claudia Cohen Center for Reproductive Medicine, Weill Cornell Medicine, New York, NY, USA; Ronald O. Perelman and Claudia Cohen Center for Reproductive Medicine, Weill Cornell Medicine, New York, NY, USA

**Keywords:** 3D culture, spherification, *in vitro* spermatogenesis, neogametogenesis, *de novo* gametes

## Abstract

In regenerative medicine, several attempts have been made to produce functional *de novo* gametes from mouse embryonic stem cells by utilizing three-dimensional (3D) culture systems. We attempted to perform neogametogensis in a novel 3D niche to generate neo-gametes ready to be used for insemination. Mouse embryonic stem cells were initially cultured on a gelatin-coated 6-well plate with a monolayer of fibroblasts before being encapsulated in sodium alginate spheres. These spheres were then immersed in specially formulated epiblast-like cell followed by primordial germ cell-like cell medium to promote differentiation into germ-like cells. Over the course of differentiation, immunofluorescence analysis revealed consistent expression patterns of spermatogenic markers. Cells were assessed for DAZL (early germ cell marker) VASA (spermatocyte), BOULE (post-meiotic) and acrosin (spermatid). The differentiated cells were then injected into oocytes and activated by calcium ionophore. Embryo development was monitored via time-lapse microscopy. Spherified neogametes on D(day)22, 29, and 36 achieved fertilization rates of 61.1%, 82.7%, and 80.0%, respectively, and blastulation rates of 20.5%, 36.0%, 26.3%, respectively. Controls exhibited fertilization rates of 89.1% and blastulation rates of 76.3%. When embryo morphokinetics were considered, D29 embryos reached compaction at 64 h and blastulation at 77 h, mirroring closely the control’s timing of 63 and 77 h, respectively. D22 embryos displayed the most delayed embryo development, of which compaction occurred at 68 h and blastulation at 106 h. Despite normal fertilization and successful blastulation, the efficiency rate remained below optimal levels. Nevertheless, our system could produce viable offspring, demonstrating that replicating the 3D seminiferous tubule environment is crucial for generating artificial gametes. This eliminates the need for allogenic/xenogenic transplantation in experimental animals.

## Graphical abstract

**Figure:**
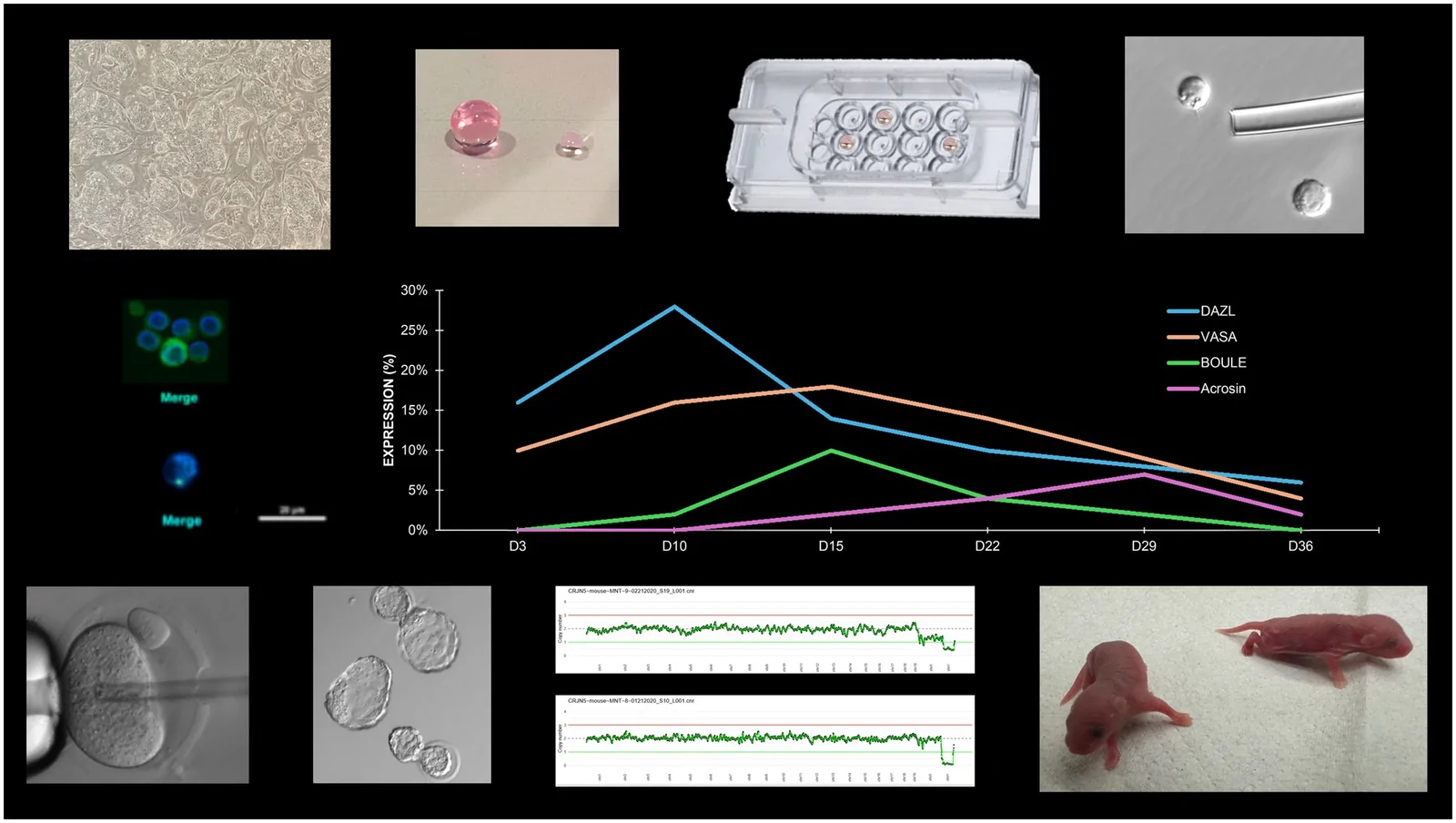
**A novel and uncomplicated three-dimensional niche accommodating complete spermatogenesis *in vitro* from mouse embryonic stem cells, resulting in neo-gametes capable of supporting pre- and post-implantation development**.

## Introduction

Infertility affects 8–12% of couples of reproductive age worldwide, with male factors accounting for 30% of these cases (Vander Borght and Wyns, 2018). ICSI can address most cases of male factor infertility, including azoospermia, provided that viable spermatozoa are surgically retrieved (Schlegel, 1999; Palermo *et al*., 2014). While surgical sperm retrieval is typically successful in cases of obstructive azoospermia, it remains unpredictable in nonobstructive azoospermia (Aizer *et al*., 2026), in which the likelihood of retrieving injectable male gametes reaches, at best, about 60%, even when microscopic testicular sperm extraction is utilized (Agarwal *et al*., 2021; Ng *et al*., 2024).

Various strategies have been explored to enhance sperm retrieval outcomes in nonobstructive azoospermia (NOA), such as treatments aimed at normalizing hormone levels to promote spermatogenesis (Hussein *et al*., 2013; Kobori *et al*., 2015; Rose *et al*., 2024). However, these treatments have had limited success in cases of spermatogenic arrest or germ cell aplasia (Ghanami Gashti *et al*., 2021), despite extensive search by several embryologists (Palermo *et al*., 2014; Brannigan *et al*., 2024).

When no spermatozoa are identified, couples are guided to use donor spermatozoa, which hinders their chances of generating their own genotyped progeny (Eisenberg, 2023; Brannigan *et al*., 2024; Han and Brannigan, 2008). Alternatively, generation of *de novo* gametes from embryonic stem cell has been explored. Over the last decade, research on neogametogenesis has largely been conducted on female reproductive cells to mitigate impaired oocyte reserves and aneuploidy. Protocols have demonstrated that functional oocytes can be generated from mouse pluripotent stem cells by coculturing them with embryonic gonadal somatic cells to produce fertilizable oocytes *in vitro* (Hayashi and Saitou, 2013; Yoshino *et al*., 2021). An extension of this work has allowed the generation of living offspring (Hikabe *et al*., 2016; Hamazaki *et al*., 2021). Other authors have offered a different perspective focusing on utilizing the somatic cell nuclei of infertile individuals, whether cumulus or endometrial stromal cells, to generate functional oocytes achieving live mouse offspring (Lee *et al*., 2022; Setton *et al*. 2023; Marti Gutierrez *et al*. 2025).

Focusing on the male gametes, in an attempt to increase the number of male reproductive cells, an individual spermatozoon directly injected into an enucleated oocyte can replicate, in an embryo-like fashion, generating haploid blastomeres that function as putative male gametes and live offspring (Takeuchi *et al*., 2009; Xie *et al*., 2025). More ambitious, however, is the ability to generate *de novo* gametes from patient-derived stem cells or induced pluripotent stem cells, particularly in men with azoospermia and Sertoli cell-only syndrome (Tsai *et al*., 2000; Robinson *et al*., 2023). To address this issue, early studies proposed a brief differentiation step to coax pluripotent stem cells into early spermatogonia. To further progress toward germ cell differentiation and spermiogenesis, these cells were cultured in the seminiferous tubules of busulfan-treated host severe combined immunodeficient mice (Reis *et al*., 2000; Liang et al. 2022). This approach has also been successful when allogeneic transplantation of spermatogenic cells was performed in mice (Brinster and Avarbock, 1994; Kanatsu-Shinohara *et al*., 2022) and nonhuman primates (Hermann *et al*., 2012; Fayomi *et al*., 2019). However, this grafting approach presents some concerns related to eventual cross-species/individual contamination, potential tissue rejection, and obvious ethical implication (Rovó *et al*., 2006; Pelzman *et al*., 2020).

A more straightforward approach would be to attempt differentiation at different spermatogenic stages *in vitro*. To pursue this route, a three-dimensional (3D) culturing system, organotypic testicular organoids have been proposed to generate germ cells retrieved from murine, porcine, and human tissue, in microwells (Sakib *et al*., 2019). Refinements in this technique have enabled the generation of mature mouse spermatozoa (Sato *et al*., 2013; Delessard et al., 2022). In addition, long-term 3D culture allowed the development of a microfluidic system in a mouse model that could generate fertile spermatozoa and yield offspring (Komeya *et al*., 2016). Recently, a more radical system utilizing 3D bio-printed scaffolds to differentiate patient-derived induced pluripotent stem cells into early germ cells was proposed (Robinson *et al*., 2022). However, these innovative culture platforms, which can achieve male germ cell differentiation, are difficult to maintain and provide inconsistent results (Mecca *et al*., 2024).

Our study proposes a new 3D niche that allows mouse embryonic stem cells (mESC) to differentiate into *de novo* gametes in an inexpensive and highly dynamic manner. The *in vitro* male germ cell differentiation sequence was followed by stage-specific and genetic/epigenetic biomarkers to depict different spermatogenic stages and was compared to the physiological timeline of mouse spermatogenesis. To demonstrate the ability of the derived germ cells to function as gametes, putative spermatid-like cells were injected into mouse oocytes and assessed throughout preimplantation morphokinetics in comparison to the physiological control.

## Materials and methods

### Study design

In this study, we investigated spermatogenic development of mESC in a 3D niche. Subsequently, we assessed murine-specific spermatogenetic markers that demonstrated protein localization and day-specific development. The ploidy of these primordial germ cell-like cells (PGCLCs) was determined by fluorescent *in situ* hybridization (FISH). These PGCLCs were enzymatically detached on D(day)22, D29, D32, and D36 and prepared for piezo-actuated intracytoplasmic injection into B6D2F1 mouse oocytes. The morphokinetics and development of these embryos were analyzed via time-lapse microscopy with distinctions drawn at the cleavage, 8 cell, morula/compaction, blastocyst, and hatching stages. Both PGCLC-derived hatching blastocysts and control were transferred to pseudo-pregnant mice. This project was approved by the IACUC of Weill Cornell Medical College under protocol number 0605-493A.

### Mouse embryonic stem cell propagation

Irradiated mouse embryonic fibroblasts (mEFs; Gibco, Thermo Fisher, Fair Lawn, NJ, USA) were prepared as previously described (Tamm *et al*., 2013). In brief, cryopreserved mEFs were placed in 37°C water bath, gently swirled, and washed with 70% methanol (Thermo Fisher). Sterilized (0.1 µm) mEF medium containing 85% Dulbecco’s Modified Eagle Medium (DMEM; Corning, Corning, NY, USA), 15% Fetal Bovine Serum (FBS; ATCC, Manassas, VA, USA), and 100 IU/ml of Penicillin–Streptomycin (Thermo Fisher) was added dropwise to the vial and mixed with 3 ml transfer pipettes (Avantor VWR, Radnor, PA, USA). The sample was centrifuged (200*g*, 5 min), the supernatant was aspirated, and the pellet was resuspended in media. mEFs were plated on 6-well gelatin-coated plates (Corning) at a density in accordance with previous studies (Jozefczuk *et al*., 2012) (56 000 cells/cm^2^).

Once fibroblasts were confluent and elongated, C57BL/6 mESC (ATCC) were thawed and processed similarly to mEF with changes being made to plating density, 1.2 × 10^4^ cells/cm^2^, and media composition: 85% DMEM, 15% FBS, 0.055 mM 2-mercaptoethanol (Thermo Fisher), 100 IU/ml Penicillin–Streptomycin (Thermo Fisher), and 1000 IU/ml ESGRO^®^ Recombinant Mouse LIF Protein (LIF; Sigma-Aldrich, St. Louis, MO, USA). Post-centrifugation of the mESC required the pellet to be gently triturated 2–3 times with 100–1000 µl pipette (Rainin, Oakland, CA, USA), ensuring clumps were still present. The medium was changed every day.

### mESC spherification

Spherification medium was curated via the addition of 0.55 g sodium alginate (Modernist Pantry, Eliot, ME, USA) to 50 ml of DMEM. This solution is kept overnight between 2 and 8°C to promote proper consistency.

Once 2 wells (9.6 cm^2^ each) of mESC reached 70–80% confluency, mESC medium was removed, and 2 ml of Trypsin–EDTA 0.25% (TE; Gibco, Thermo Fisher) was added. The plate was incubated (37°C, 5.5% CO_2_) for 10–15 min, facilitating enzymatic activity. The cells were aspirated and diluted in mESC medium containing FBS to neutralize trypsin activity. The solution was then centrifuged (200*g*, 5 min), the supernatant was removed, and the pellet was resuspended in 2 ml of mESC media (1.5 × 10^6^ cells/ml). Concurrently, 0.125 g of calcium chloride (Modernist Pantry) was combined with 25 ml of DMEM in a 50-ml beaker.

Refrigerated spherification media and mESC (1.5 × 10^6^ cells/ml) were combined in a 9:1 fashion, mixed, and using a 100–1000 μl tip (Rainin) this cell and sodium alginate mixture was added dropwise to the calcium chloride beaker (Supplementary Video S1). Spheres were then formed via a double replacement reaction (Hosokawa *et al*., 2024), with the production of calcium alginate and sodium chloride, creating gelatinous polysaccharide spheres. The spheres were then equally distributed into 4-well dishes (Thermo Fisher) until all mESC were used (Supplementary Fig. S1). The initial sphere concentration ranged from 1.0 × 10^4^ to 2.0 × 10^4^ cells/sphere.

### Cell differentiation in spheres

Spheres were initially submerged in epiblast-like cell (EpiLC) medium consisting of 0.02 µg/ml Activin A recombinant protein (Thermo Fisher), 0.012 µg/ml bFGF protein (Thermo Fisher), 1% KnockOut™ Serum Replacement (KSR; Thermo Fisher) for 3 days (Hayashi *et al*., 2011; Li *et al*., 2019), media were changed every day.

By day 3, both cultures were immersed in PGCLC media, constituting: 15% KSR, 1% MEM Non-Essential Amino Acids (Thermo Fisher), 1 mM sodium pyruvate (Thermo Fisher), 0.055 mM 2-mercaptoethanol, 100 IU/ml Penicillin–Streptomycin, 2 mM l-glutamine, 500 IU/ml LIF, 100 ng/ml of mouse BMP-4 recombinant protein (Thermo Fisher), 100 ng/ml of mouse SCF recombinant protein (Thermo Fisher), 50 ng/ml of mouse EGF recombinant protein (Thermo Fisher), and 50 µM of retinoic acid (Sigma-Aldrich) (Hua *et al*., 2009; Hayashi *et al*., 2011). The spheres were kept submerged in the PGCLC medium until D40 or until they were broken down, with the culture medium replaced on alternating days.

### Differentiated cell extraction

Spheres were sampled on day 3 (during EpiLC differentiation) and on days 3, 10, 15, 22, 29, 32, and 36 (during PGCLC differentiation). Stage-specific markers and ploidy status were investigated. Immunofluorescence and FISH were performed on these days, and the cells were injected on D22, D29, D32, and D36. For each sampling cycle, ∼16 spheres (one plate) were isolated, placed into a 15-ml falcon tube, and mechanically broken down using a spatula tip (Corning). The spread cell alginate was mixed with 5 ml of TE, gently pipetted, and incubated for 5–10 min. Basal medium containing FBS was added, and the solution was centrifuged at 200*g* × 5 min. For oocyte injection, cells were resuspended in 100 µl of PGCLC media and 8 µl was allocated to an ICSI dish (Thermo Fisher). For immunofluorescence and FISH, pelleted cells were resuspended in 10 ml of PBS, mixed thoroughly, centrifuged (200*g*, 5 min), and resuspended in 100 µl of PBS. The cells were then smeared onto super frost slides (Thermo Fisher).

### Cell characterization

Stage-specific cellular biomarkers were used to determine neogametogenesis progression. The post-extraction cell concentration for the spheres was ∼0.5–1 × 10^5^ cells/ml. The smeared super frost slides were left to dry overnight, fixed in 4% paraformaldehyde solution (15 min) and then washed thrice in phosphate buffered saline (PBS). The following day, slides were permeabilized (10 min) in 0.1% Triton X-100 in PBS and washed with PBS. Once dried, 50 µl Pierce blocking buffer (Waltham, MA, USA) was added to each, left to incubate for one hour, and washed again. An aliquot of primary antibodies (50 µl); DAZL (Abcam, Cambridge, UK; ab34139), DDX4 (VASA) (Abcam, ab13840), BOULE (Abcam, ab155027), and acrosin (Abcam, ab203289) were smeared onto each slide, incubated in the fridge overnight (2–6°C), and washed in PBS. Fifty microliters of Alexa Fluor secondary antibody (Abcam, ab150077) was added. The slides were incubated at room temperature for 2 h and then washed. To visualize the cell nuclei, DAPI antifade (Sigma-Aldrich) was added and 100 cells were assessed for the presence of each biomarker.

### Cytogenetics

FISH was used to determine the ploidy of the extracted cells. Aliquots (10 μl) of cells extracted on days 22, 29, 32, and 36 were smeared onto pre-cleaned glass slides and air-dried overnight. Slides were fixed in Carnoy’s fixative (3:1 methanol:acetic acid) for 15 min at room temperature and incubated in a 37°C slide moat overnight. Slides were dehydrated in an ethanol series (70%, 85%, and 100%; 2 min each) and air-dried. A hybridization mixture containing probes specific for chromosomes X, Y, 16, and 18 (Empire Genomics, Buffalo, NY, USA) was applied to each slide, which was then coverslipped and sealed with rubber cement. Hybridization was carried out at 73°C for 2 min, followed by 37°C for 16 h. The cell nuclei were counterstained with DAPI and visualized using an Olympus BX61 fluorescence microscope at 1000× magnification (Applied Imaging, CytoVision v3.93.2). The incidence of haploidy and diploidy was assessed in at least 500 cells per sample and compared to mESC controls. Each sample was also processed and assessed in replicates to maintain a 2–3% FISH error (Cheung *et al*., 2019).

### Gamete preparation

B6D2F1 mice (8–12 weeks old, Jackson Laboratory, Farmington, CT, USA) were stimulated using 0.1–0.2 ml of ready-to-use pregnant mare serum gonadotropin/inhibin superovulation reagent (CARD HyperOva^®^, Cosmo Bio, Japan). Forty-eight hours after intraperitoneal injection, 7.5 IU of hCG (Sigma-Aldrich) was used to trigger oocyte maturation. Mice were euthanized by cervical dislocation ∼16 h post-hCG stimulation, and their oviducts were dissected to collect cumulus–oocyte complexes (COCs). COCs were extracted from the ampullae of the oviducts and treated with 40 IU/ml hyaluronidase (Origio, CooperSurgical, Trumbull, CT, USA; Cumulase), after which they were denuded using a microcapillary. Oocytes were serially washed thrice in potassium simplex optimized media (KSOM, Cosmo Bio, Japan), incubated (37°C, 5% CO_2_, 90% relative humidity (RH)), and left in equilibrated KSOM for use in subsequent steps.

Mature spermatozoa for control were collected from euthanized male B6D2F1 mice (Jackson Laboratory). The lower abdomen was sanitized with betadine, an incision was made to access the cauda epididymis, and the excised tissue was placed in human tubal fluid medium (Cosmo Bio, Japan). The spermatozoa were incubated for at least 2 h and then diluted to 3 million/ml for piezo ICSI (Setton *et al*., 2023).

### Intracytoplasmic cell injection and assisted gamete treatment

Mouse metaphase II oocytes were injected with cells extracted from both 3D and 2-dimensional cultures. Piezo-ICSI was performed as previously described (Yoshida and Perry, 2007). Briefly, the injection pipette was first back-loaded with Fluorinert (Sigma-Aldrich), cells were subsequently aspirated into the injection pipette, and then moved to the egg droplet. A holding pipette was used to stabilize the oocyte with the polar body at the 12 or 6 o’ clock position. A piezo pulse created a break in the zona, allowing the injection pipette to pass through and place the cells at the 9 o’clock position. Injected oocytes were exposed to 50 μM calcium ionophore in G-TL medium (Vitrolife, Göteborg, Sweden) for 10 min at 37°C and then rinsed and placed in fresh G-1 medium (Cheung *et al*., 2020). The cell-injected embryos were transferred to EmbryoSlides (Vitrolife) and monitored using a time-lapse microscope (Vitrolife).

As a positive control, motile spermatozoa were placed in 7% polyvinylpyrrolidone (Irvine Scientific, Santa Ana, CA, USA), mechanically decapitated, and injected in the standard fashion (Yoshida and Perry, 2007). Injected oocytes and resulting embryos were cultured and monitored as described above.

### Ploidy study on the conceptuses

A blastomere biopsy was performed on some embryos at the 8-cell stage for aneuploidy assessment. Whole genome amplification was performed on the samples using the SurePlex DNA Amplification kit (Illumina, San Diego, CA, USA), and next-generation sequencing (NGS) was used to identify chromosomal gain(s) and/or loss(es), as well as to detect mosaicism.

### Embryo transfer and pregnancy assessment

Hatching blastocysts were collected and transferred into the uterine horn of 2.5-day-post-coitus (dpc) psuedopregnant CD-1 recipient mated with vasectomized CD-1 male. Briefly, 2.5 dpc surrogate mice were anesthetized and placed on a heated stage. A laparotomy was performed to expose the uterine horn. The uterine horn was secured with a Serrefine clamp, and a small puncture was made by a needle. A glass capillary carrying the blastocysts was then inserted into the uterine cavity via the puncture. The blastocysts were then released into the uterine cavity. The uterine horn was then returned to the abdominal cavity. Surgical sites were closed via suture and skin closed by wound clip. The mice were given standard post-surgery care and monitored for signs of pregnancy by abdominal palpation. Pregnant mice were allowed to deliver naturally.

### Statistical analysis

A statistical *t*-test was performed to compare the clinical outcomes between the control group and each of the injection days.

*A priori* power analysis was performed for different effect sizes to validate the calculated *post hoc* power. The calculation was performed using G*Power software (Heinrich-Heine-Universität Düsseldorf, North Rhine-Westphalia, Germany).

## Results

### Assessment of *in vitro* spermatogenesis

Differentiation of mESC was tracked in 3D cultures and verified using numerous methodologies. Baseline and time-interval sampling for biomarkers were performed according to the length of differentiation. This analysis was conducted using mESC negative controls, yielding 0% DAZL, 1% VASA (DDX4), 0% BOULE, and 0% acrosin expression. Mouse testicular tissue was used as a positive control, as described in the Materials and Methods section.

### Immunofluorescence

**Figure 1.**
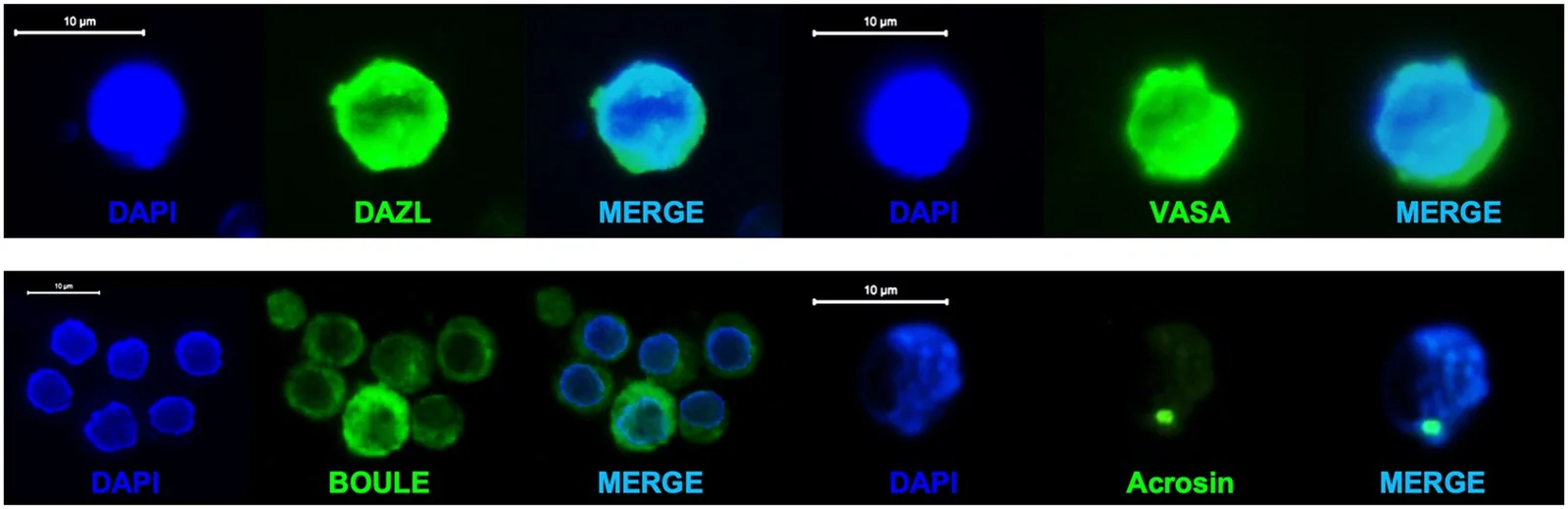
**Markers of spermatogenesis.** Immunofluorescence images show localization patterns for spermatogenic biomarkers in differentiated mouse ESCs extracted from calcium alginate spheres. DAZL, VASA, and BOULE demonstrated cytoplasmic expression, whereas acrosin demonstrated focal intracellular staining. DAPI was used to visualize nuclei, and merged images show the overlap between nuclear and biomarker staining. Scale bars represent 10 µm. DAZL, deleted in azoospermia-like; VASA/DDX4, DEAD-box helicase 4; BOULE, boule homolog; DAPI, 4′,6-diamidino-2-phenylindole.

**Figure 2.**
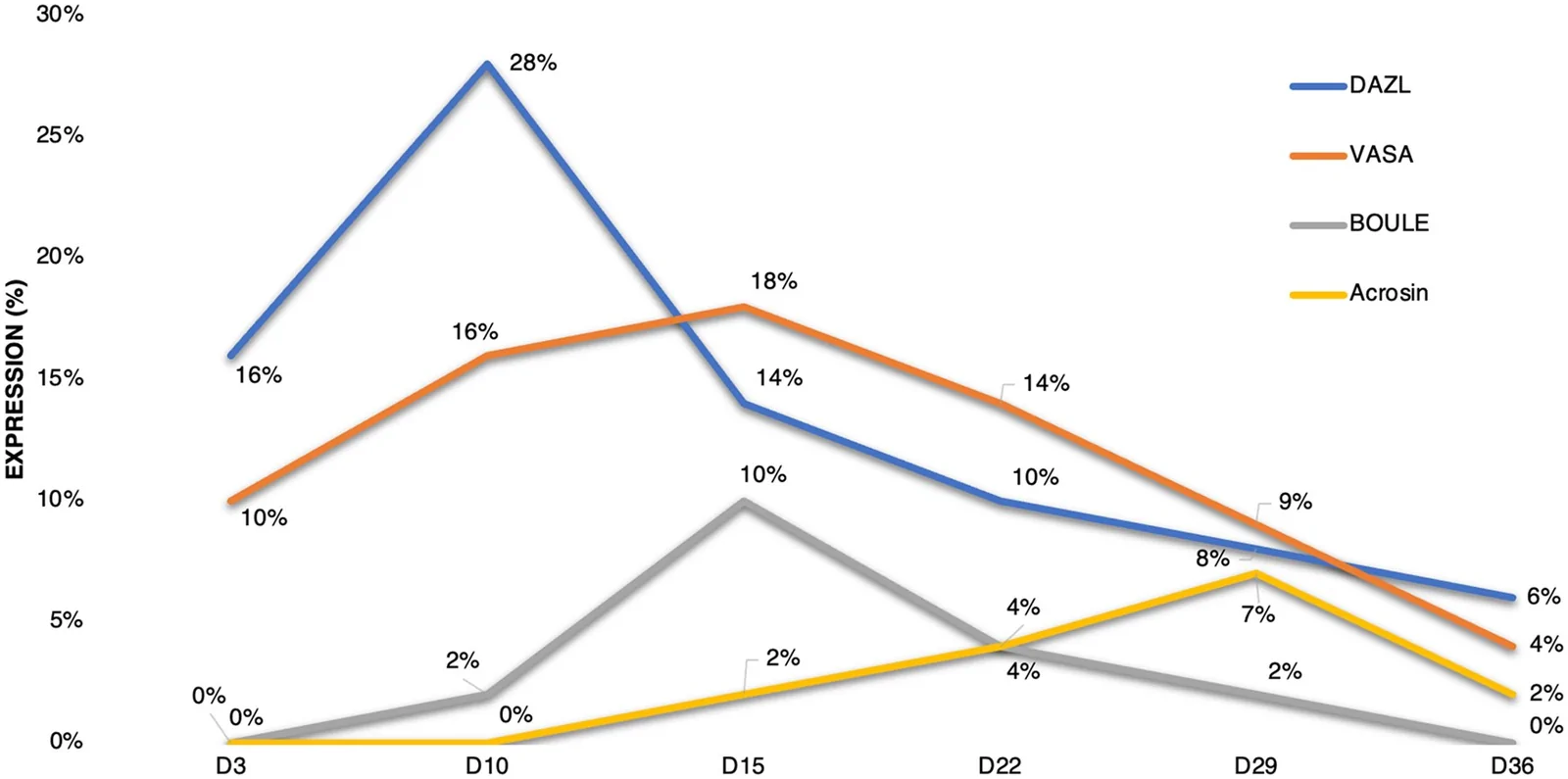
**Immunofluorescence testing for spermatogenic markers.** Quantification of spermatogenic biomarker expression (% cells) is shown across defined intervals in sphere culture. Stage-associated biomarker expression was monitored by immunofluorescence during differentiation. Acrosin expression peaked at D29, corresponding to the highest observed expression of this spermatid-associated marker. D, day.

Immunofluorescence revealed notable patterns (Fig. 1) in the expression of spermatogenic markers across different developmental stages and conditions (Fig. 2). On day 3, cells differentiated on plates showed an expression of DAZL of 29% and a low expression of VASA of 1%. DAZL, an early spermatogonial marker, was expressed at 16% in spheres, whereas VASA, a marker associated with further progression in spermatogenesis, was expressed at significantly higher levels with an expression of 10% (*P* <0.001). This suggests that the culture conditions in spheres are more suitable for spermatogenesis.

By day 10, the expression levels of VASA and DAZL in cells differentiated on plates had increased with VASA at 7% and decreased with DAZL at 23%. BOULE, a marker specific to postmeiotic spermatocytes, was also expressed in 0.5% of plate-differentiated cells. In contrast, spheres exhibited higher expression of these markers, with VASA at 16%, DAZL at 28%, and BOULE at 2% (*P* <0.001). The increase in the expression of these markers indicates that sphere culture conditions support ongoing spermatogenic development and advancement to more advanced stages better than traditional plate cultures.

Under sphere culture conditions, the cells were assessed for differentiation on days 15, 22, 29, and 36. On day 15, DAZL expression was 14%, and VASA expression was 18%. BOULE expression was 10% and acrosin, a marker for spermatids, had an expression of 2%. The presence of BOULE and acrosin reflects the maturation of germ cells into the later stages of spermatogenesis within the spheres.

On day 22, VASA and DAZL expression decreased to 14% and 10%, respectively, while acrosin rose to 4%. This indicated the continued maturation of spermatogenic cells into late-stage germ cells.

By day 29, VASA expression slightly decreased to 9%, BOULE dropped to 2%, and acrosin increased to 7%. The increase in acrosin and consistent expression of VASA suggest that spermatogenesis within the spheres reaches a more advanced stage, with a predominance of early and mid-stage germ cells.

On day 36, DAZL expression was 6%, while BOULE had decreased to 0%. Interestingly, comparing to day 29, acrosin expression decreases to 2%. The decline in BOULE and acrosin levels at this stage might reflect a transition out of the period of peak spermatogenesis, which normally lasts for 34 days *in vivo*.

### Ploidy assessment of the neogametes

**Figure 3.**
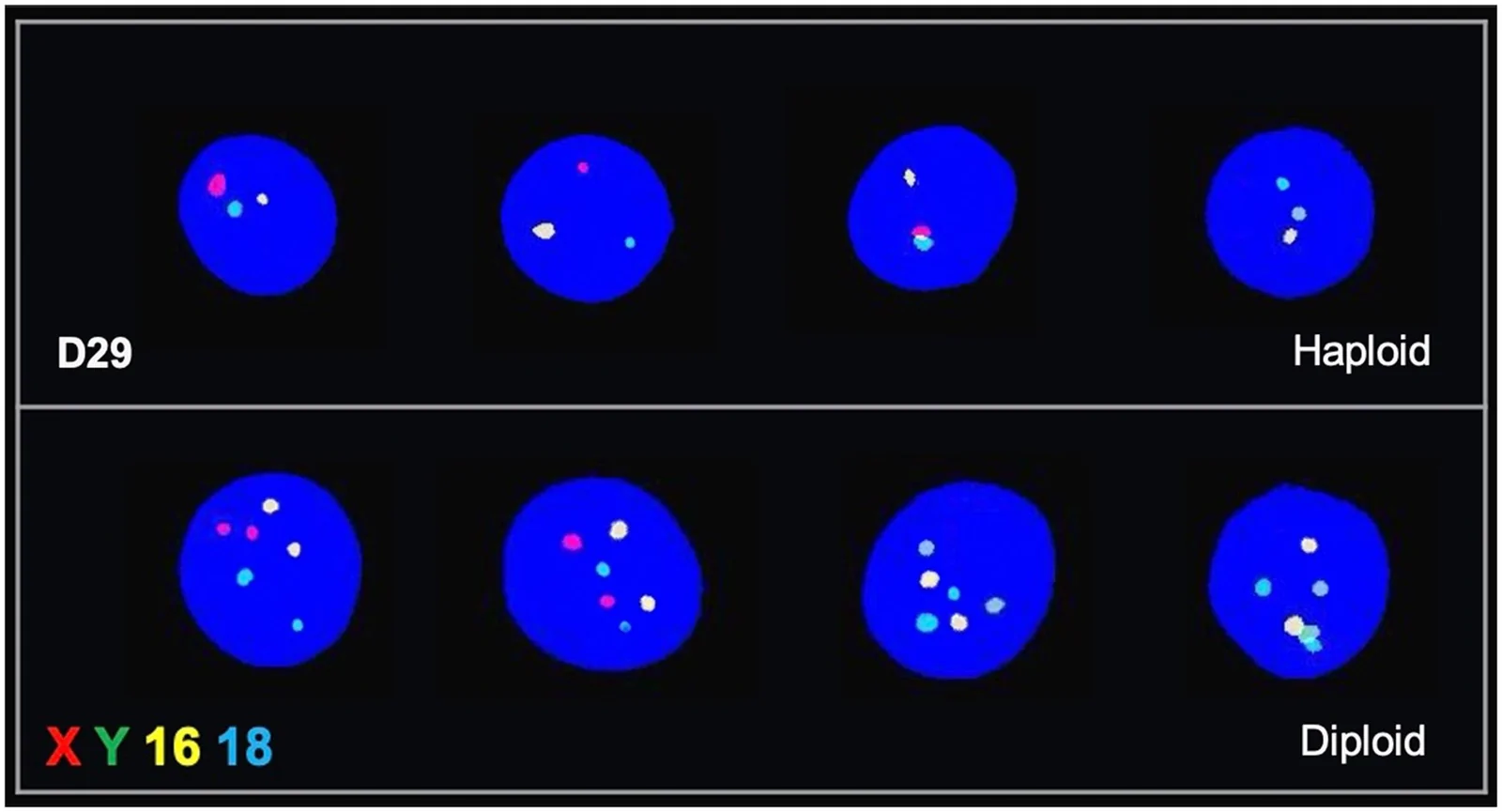
**Cytogenetics.** Cytogenetic analysis was used to monitor meiotic progression during differentiation. Initially, mouse embryonic stem cells (mESCs) were diploid, as shown in the bottom half of the panel. By D29, over 80% of the cells extracted from spheres were haploid, confirming that the cells successfully underwent meiotic progression. Red, chromosome X probe; green, chromosome Y probe; yellow, chromosome 16 probe; blue, chromosome 18 probe; D, day.

FISH analysis of the extracted cells was performed at weekly intervals to monitor the progression toward meiotic completion. No haploid cells (0/257) were observed at the onset of differentiation. However, the proportion of haploid cells increased over time, rising from 25.7% (55/214) on day 10 to 33.7% (70/208) on day 15, 57.9% (128/221) on day 22, and finally 80.6% (83/103) on day 29 (*P* <0.02) (Fig. 3).

### Embryo development competence of the male gametes

The controls achieved a fertilization rate of 89.1% (335/376), with 87.8% (330/376) reaching the 2-cell stage, and 83.8% (315/376) of these developing to the 8-cell stage. Only 76.3% (287/376) of these embryos formed blastocysts and 75.4% (284/376) hatched. A total of 132 hatching control blastocysts were transferred into 23 recipients. Of those, 18 were confirmed pregnant and naturally delivered 77 healthy offspring with mean birth weight of 1.63 ± 0.2 g.

D22 cells had a lower fertilization rate of 61.1% (165/270), leading to 59.9% (162/270) reaching the 2-cell stage and 25.3% (69/270) developing to the 8-cell stage. Only 20.5% (56/270) of these embryos made it to blastocysts and 18.6% (50/270) hatched.

D29 cells achieved a comparable fertilization rate of 85.9% (341/397), which led to 84.6% (336/397) reaching the 2-cell stage, and 41.3% (164/397) developing to the 8-cell stage. Of these, 36.8% formed blastocysts (146/397) and 34.8% (138/397) hatched.

**Figure 4.**
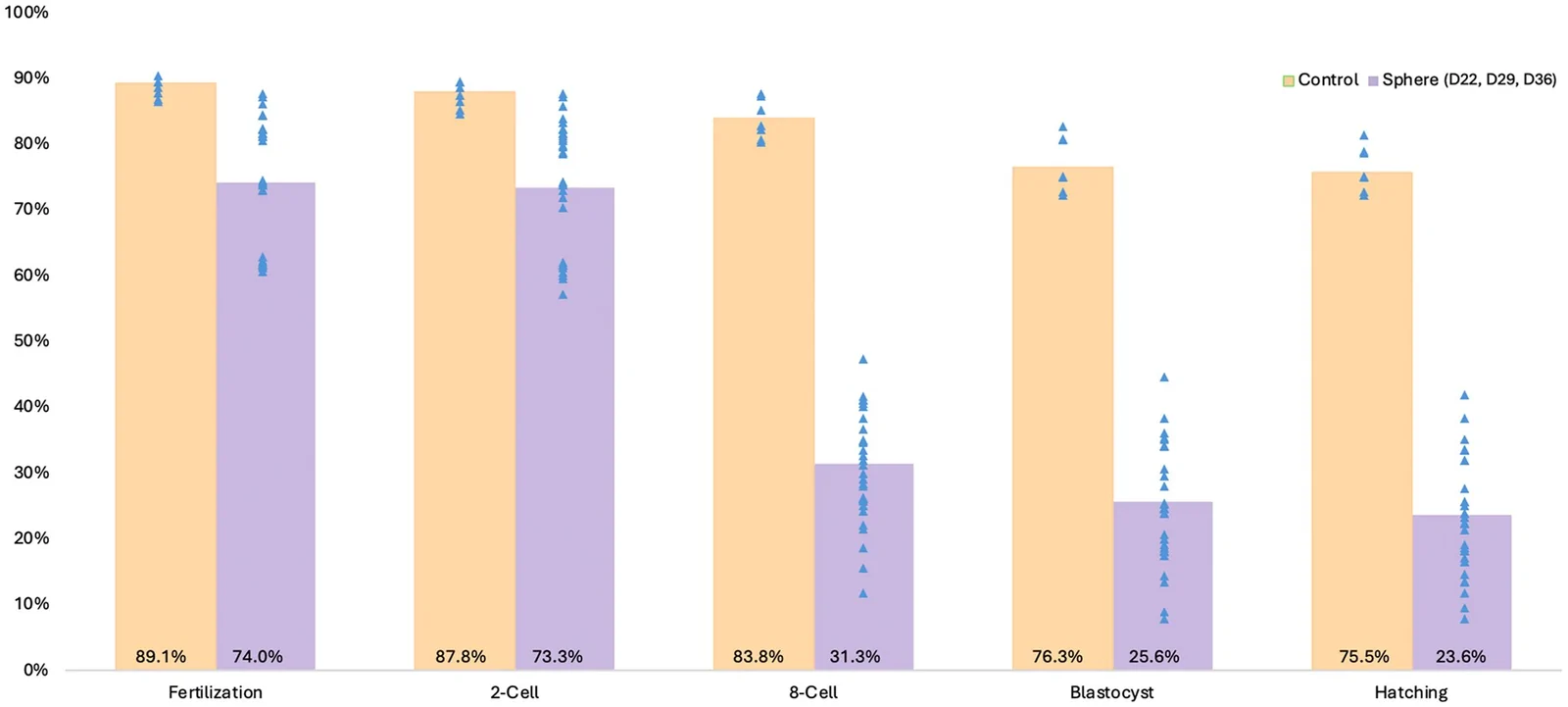
**Fertilization and embryo development.** Fertilization and preimplantation embryo development were compared between control spermatozoa-derived embryos and embryos generated using cells extracted from D22, D29, and D36 spheres. The individual points show the distribution of results from experimental replicates. Fertilization and 2-cell development were comparable between groups, whereas embryos generated from sphere-derived cells showed reduced development beyond the 2-cell stage. D, day.

D32 cells yielded a lower fertilization rate of 80.0% (219/274), with 79.3% (217/274) reaching the 2-cell stage, and 32.0% (87/274) developing to the 8-cell stage. Of these embryos, 26.3% (72/274) developed into blastocysts and 24.4% (67/274) hatched (Table 1, Fig. 4).

**Table 1.** Fertilization and embryo development at each injection day compared to the control.

	Control	D22	D29	D36
**Total oocytes injected**	376	270	397	274
**2PN**	335 (89.1)	165 (61.1)	341 (85.9)	219 (80.0)
**2-cell**	330 (87.8)	162 (59.9)	336 (84.6)	217 (79.3)
**8-cell**	315 (83.8)	69 (25.3)	164 (41.3)	87 (32.0)
**Blastocyst**	287 (76.3)	56 (20.5)	146 (36.8)	72 (26.3)
**Hatching**	284 (75.4)	50 (18.6)	138 (34.8)	67 (24.4)

Fertilization and preimplantation embryo development were assessed after injection with control spermatozoa or cells extracted from D22, D29, and D36 spheres. D29 showing the highest rates across all developmental outcomes and fertilization and 2-cell development rates closest to the control group. Values are presented as n (%), with percentages calculated using the total number of injected oocytes as the denominator. D, day; 2PN, two pronuclei.

A total of 98 blastocysts from experimental cohort were transferred into 19 recipient pseudopregnant mice. To date, only one pregnancy was observed, followed by the delivery of two live male pups, with birth weight at 1.45 and 1.61 g.

### Conceptus morphokinetic characteristics

Fertilized oocytes from the control group reached the 2-cell stage (*t*_2c_) at 17 h after injection. They then developed to the 4-cell stage (*t*_4c_) 22 h later and 8-cell stage (*t*_8c_) after a further 13 h. This was followed by compaction (*t*_comp_) lasting 11 h and blastulation (*t*_blast_) over a further 14 h.

Following injection with cells extracted from D22 spheres, fertilized oocytes reached *t*_2c_ after 19 h, *t*_4c_ after a further 21 h, and *t*_8c_ over a further 13 h, followed by *t*_comp_ and *t*_blast_ 15 and 38 h later, respectively.

**Figure 5.**
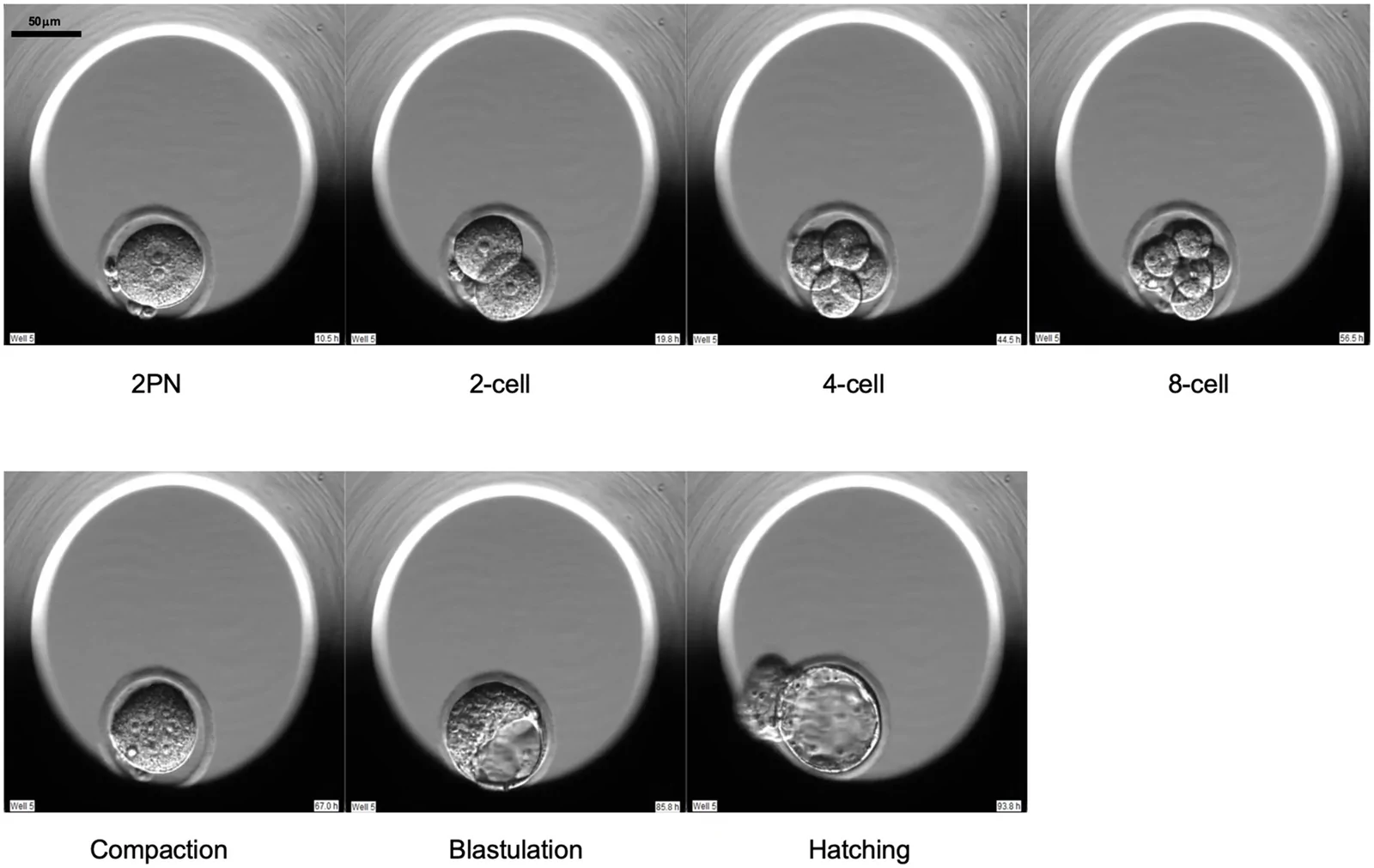
**Preimplantation embryo development.** Sequential time-lapse images show progression from the two-pronuclear stage through cleavage, compaction, blastulation, and hatching post-injection of oocyte with a D29 sphere-derived cell. D, day; 2PN, two pronuclei.

Fertilized oocytes injected with D29 cells reached *t*_2c_ after 17 h, *t*_4c_ 20 h later, and *t*_8c_ after a further 13 h, followed by *t*_comp_ and *t*_blast_ 14 and 13 h later, respectively (Fig. 5, Supplementary Video S2). The development time of this cohort was similar to that of the control group.

**Figure 6.**
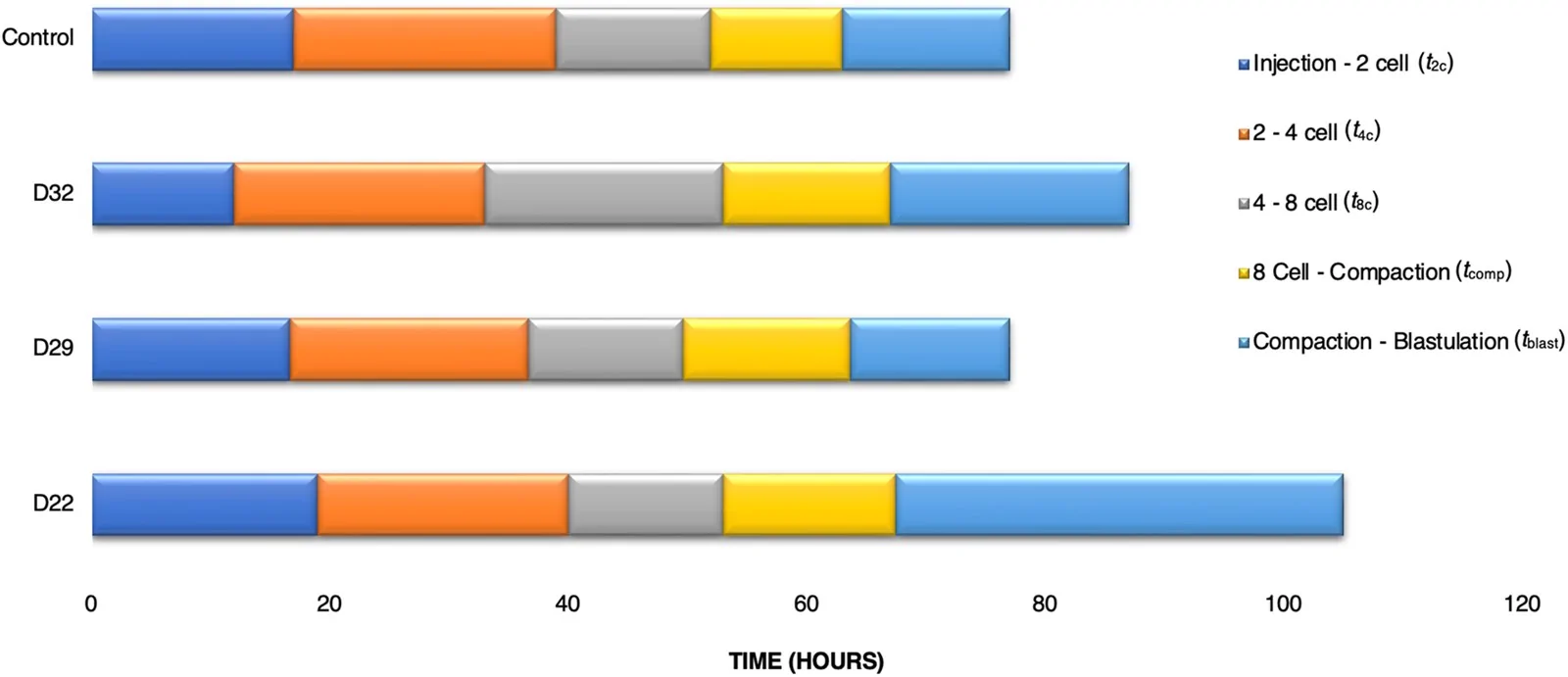
**Morphokinetics.** Time-lapse monitoring was used to compare the duration of each developmental interval in embryos generated from control spermatozoa and cells extracted from D22, D29, and D32 spheres. Embryos generated using D29 cells showed developmental timing most similar to the control group, whereas embryos generated using D22 and D32 cells showed prolonged progression to blastocyst formation. D, day.

The cells from the D31 spheres, however, had shorter *t*_2c_ and *t*_4c_ values at 12 and 21 h, respectively. After reaching *t*_8c_ (20 h later), the development was much slower than that of the control, with a *t*_comp_ of 14 h and a *t*_blast_ of 20 h (Table 2, Fig. 6).

**Table 2.** Morphokinetics analysis from time-lapse monitoring of the conceptuses.

	Injection–2 cell	2–4 cell	4–8 cell	8-cell–compaction	Compaction–blastulation
**Control**	17	22	13	11	14
**D22**	19	21	13	15	38
**D29**	17	20	13	14	13
**D36**	12	21	20	14	20

Morphokinetic development of conceptuses generated from control spermatozoa or cells extracted from D22, D29, and D36 spheres was assessed by time-lapse monitoring. Values indicate the average time, in hours, between each developmental stage. D29 conceptuses showed morphokinetic timing most similar to the control, while D22 and D36 conceptuses showed delayed progression to blastulation. D, day.

### Ploidy of the conceptuses

**Figure 7.**
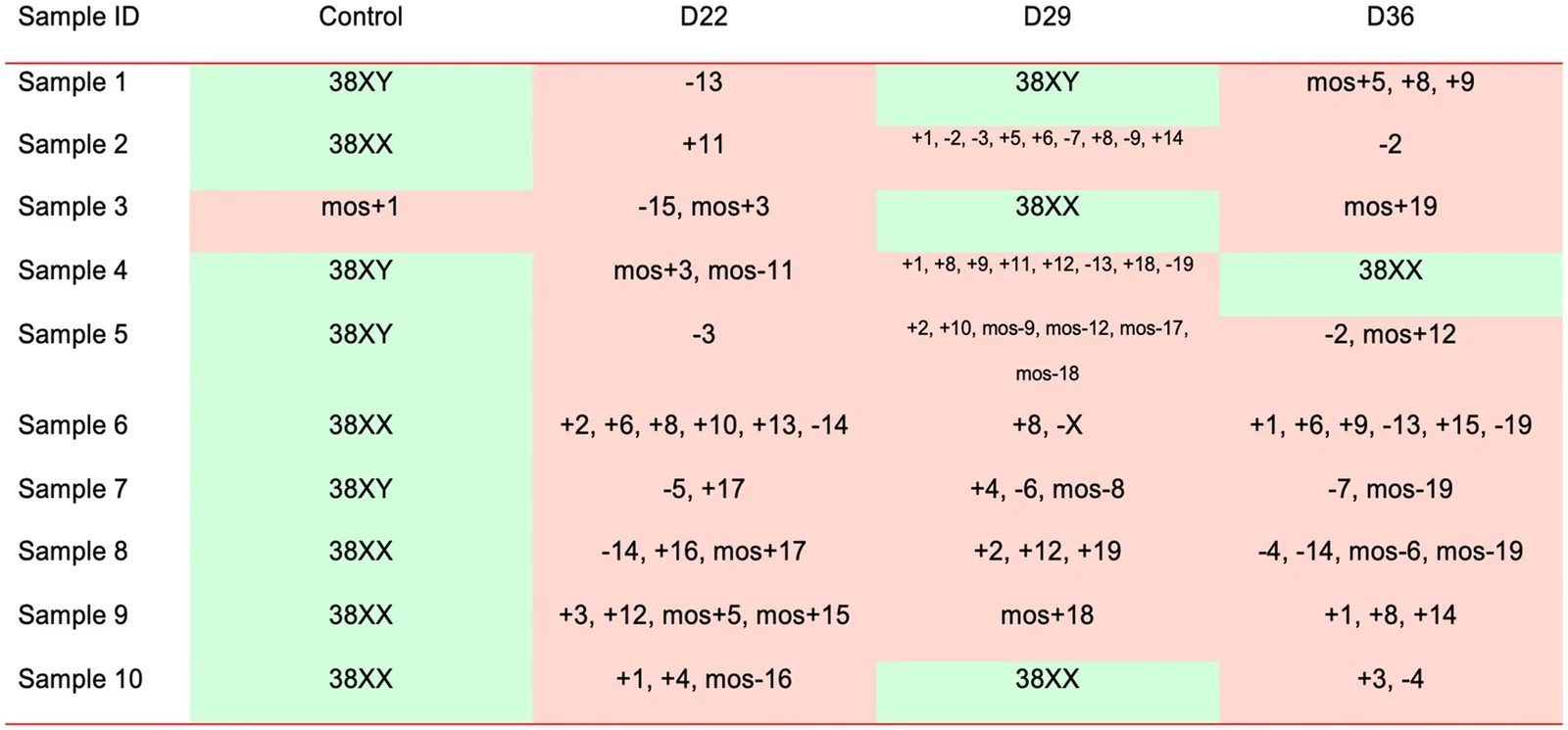
**NGS assessment of embryo ploidy.** Next-generation sequencing (NGS) was used to assess control embryos and embryos generated using cells extracted from D22, D29, and D36 spheres. Euploid embryos were identified among sphere-derived groups, including 38XY embryos (D29 Sample 1) and 38XX embryos (D29 Samples 3 and 10, D36 Sample 4). Green shading indicates euploid embryos, whereas red shading indicates embryos with chromosomal abnormalities. Aneuploid embryos showed whole-chromosome gains (+11; D22 Sample 2), whole-chromosome losses (−13; D22 Sample 1), and/or mosaic abnormalities (mos+19; D36 Sample 3). D, day; mos, mosaic; +, chromosomal gain; −, chromosomal loss.

A total of 40 embryos were assessed for aneuploidy using NGS. All the embryos tested in the D22 group were aneuploid. From the embryos tested at D29, 3/10 (30%) were euploid. Of the embryos tested on D32, only 1/10 (10%) was determined to be euploid. The aneuploidy was random, and there were no chromosomes that were repeatedly affected (Fig. 7).

## Discussion

In the present study, we established a 3D culture system capable of maintaining optimal cell growth and proliferation. This system entails the creation of a physiological semipermeable membrane capable of curtailing cells in an enclosed space, reproducing the structure of the seminiferous tubule. The 3D spheres were submerged in culture medium to ensure continuous exposure of the confined cells to a conducive environmental milieu. The direct spherification system appears to be an adequate model for neogametogenesis, allowing embryonic stem cells to undergo male germline differentiation, even without supporting cells, being solely supported by different growth factors supplied in the surrounding medium.

A similar alginate-based scaffold, loaded with sorted spermatogonial stem cell and Sertoli cells has previously been proposed to support partial germ-cell differentiation and cell organization resembling the cellular structure of the seminiferous tubules (Lee *et al*., 2001). In a more recent study, a similar concept using 3D printed alginate scaffold reported that 66% of the cell constructs reached postmeiotic male germ cell *stage*; however, the differentiation resulted in disorganized cell clusters and uneven distribution of supporting cells (Baert *et al*., 2019). Our novel system described here, using exclusively calcium alginate for the sphere, yielded 60% haploidy, of which 5% was determined to be spermatid, comparably starting from embryonic and induced pluripotent stem cells, despite the absence of supportive Sertoli cells.

Our sphere enclosure did not preclude the coating of the inner surface with laminin, thus providing a more suitable adhesion substrate for differentiating cells. By integrating laminin-521 into the spherical culture system, we enhanced the structural organization by providing cell anchorage that facilitated differentiation consistency beyond the uncoated alginate scaffolds. The laminin-coated spheres were able to achieve 82% haploidy and yielded 8% spermatids, achieving comparable results to a previous study using a laminin-coated 3D culture of mouse pluripotent stem cells coaxed toward spermatogenesis, reaching a spermatid development rate of 7.8% (Ishikura *et al*., 2021). Indeed, laminin has been shown to have a positive effect on human germ cells, facilitating anchorage and differentiation by enabling better cellular structural organization (Jabari *et al*., 2023).

Studies reporting a more sophisticated approach reported a presumptive spermatid maturation of 44% ± 31% of their testicular organoids (Richer *et al*., 2024), without clarifying the spermatid rate. Our system instead achieved an 80% haploidy rate with 7% expression of a specific spermatid marker, still without the need for supportive Sertoli cells. Another sophisticated system utilizing 3D printed scaffolds require expensive equipment and additional expertise for fabrication, resulting in cumbersome and less reproducible devices (Bashiri *et al*., 2023). In our sphere environment, because of their membrane transparency, it was possible to monitor secluded cell development by simply placing the spheres in a culture dish on an inverted microscope and culturing them in a standard incubator. The sphere system is scalable, can be sized according to the number of enclosing cells, and can be further miniaturized and mounted on a dedicated slide for continuous time-lapse observation.

In several reports describing *in vitro* spermatogenesis approaches, the last steps of spermiogenesis are carried out by transplanting differentiating cells into host organisms for allografts (Van Saen *et al*., 2009; Lim *et al*., 2014) or xenografts (Liang *et al*., 2022). This is feasible only in animals and would preclude this approach to neogametogenesis in humans. Unlike *in vivo* graft-based approaches (Yokonishi *et al*., 2013), our system is entirely *in vitro*, enabling direct visualization of cell progression and easy sampling of spheres. The shortcoming of this system is related to the fact that haploid cells are void of the acrosome and require chemicals to trigger oocyte activation. Moreover, modification of the injection technique, which requires the utilization of another compound, is needed to allow fusion between the oocyte and the neogamete. Indeed, through the adoption of these expedients, we were able to achieve a fertilization rate of 87.9%, comparable to the most successful studies reported in the literature that instead reported *in vivo* spermiogenesis ranging from 28% to 80% (Honaramooz *et al*., 2002; Schlatt *et al*., 2003; Ohta and Wakayama, 2005; Fayomi *et al*., 2019).

Although the limitations of this study may be represented by the absence of epigenetic investigation of the conceptuses, this was compensated for by the continuous time-lapse observation of the conceptuses generated by these neogametes, as well as the generation of live offspring. In contrast, recent approaches have reported embryo development only up to the 2-cell stage prior to transfer into pseudopregnant females without complete morphokinetic monitoring of the embryonic steps (Kamoshita *et al*., 2025). In our study, delayed blastulation may have been related to improper paternal imprinting of neogametes. Imprinted genes of interest include *Igf2-H19* and *Dlk1-Dio3*, given that these two control regions are essential to embryonic development, and improper methylation can be lethal (Wei *et al*., 2025). Nonetheless, we provided a cytogenetic assessment of these cells, and most importantly, a ploidy assessment by FISH. Emboldened by this initial success in the mouse system, we have also carried out preliminary experiments using human stem cells/induced pluripotent stem cells in the same sphere system and we were able to achieve 50% haploid cells that, following injection into oocytes, have yielded two euploid embryos (McCarter *et al*., 2025). This preliminary success in generating functional gametes in an entire *in vitro* system confirmed the translation of this sphere-supported neogametogenesis approach.

Our 3D culture allowed mESC to progress predictably through the expected differentiation and maturation steps. These neogametes demonstrated fertilization comparable to that of the control and sustained preimplantation embryo development, mostly indistinguishable from natural conceptuses generated from *in vivo* matured spermatozoa. The ability to replicate a 3D seminiferous tubule environment is essential for generating competent artificial gametes. This eliminates the need for allogenic/xenogenic transplantation in experimental animals, suggesting that our model is clinically feasible.

## Supplementary Material

gaag036_Supplementary_Data

## Data Availability

The data underlying this article are available in the Dryad Digital Repository at https://doi.org/10.5061/dryad.j0zpc86wh.
