# Supplementary material for: Induction of male neogametogenesis in a three-dimensional microenvironment supporting successful fertilization and proper embryo development
Source: Mol Hum Reprod. 2026 Jun 4;32(3):gaag036. doi: 10.1093/molehr/gaag036 (PMC13338338; doi:10.1093/molehr/gaag036)
Supplement: gaag036_Supplementary_Data [file gaag036_supplementary_data.zip › Supplementary Information.pdf]

Supplementary Information

Induction of Male Neogametogenesis in a Three-Dimensional Microenvironment Supporting Successful Fertilization and Proper Embryo Development

Eros Lari, Lily Ng, Philip Xie, Stephanie Cheung, Sabrina Bains, Zev Rosenwaks, Gianpiero D. Palermo

Supplementary Figure S1: A Simple Cell Culture System

Supplementary Video S1*: Spherification Technique

Supplementary Video S2*: Time Lapse of Embryo Development

*Supplementary Videos S1 and S2 have been provided as separate files.

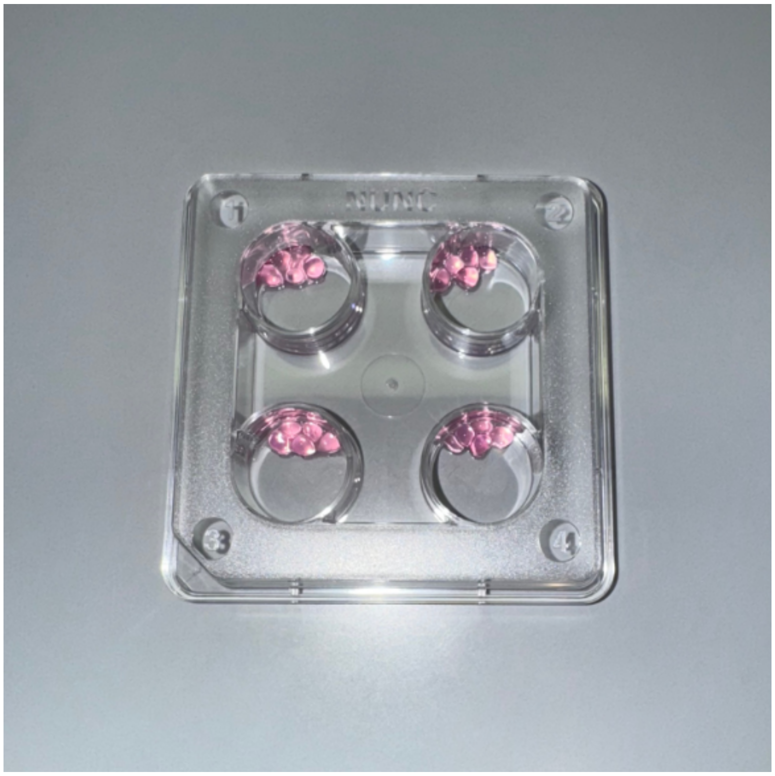

Supplementary Figure S1: A Simple Cell Culture System

Mouse embryonic stem cells (mESC) were contained in simple calcium alginate spheres and distributed into compact 4-well dishes. Culture media was added into each well to support differentiation of the neogametes.

Supplementary Video S1: Spherification Technique

Spherification media containing sodium alginate combined with mouse embryonic stem cells (mESC) was added dropwise to calcium chloride solution to produce the simple three dimensional niche.

Supplementary Video S2: Time Lapse of Embryo Development

Following injection of day 29 cells into oocytes, the developing conceptuses were monitored by time lapse. The development of the neogamete closely mimicked a control oocyte injected with a natural spermatozoon.
