# Supplementary figures and images for: Induction of male neogametogenesis in a three-dimensional microenvironment supporting successful fertilization and proper embryo development
Source: Mol Hum Reprod. 2026 Jun 4;32(3):gaag036. doi: 10.1093/molehr/gaag036 (PMC13338338; doi:10.1093/molehr/gaag036)

## Slide 1
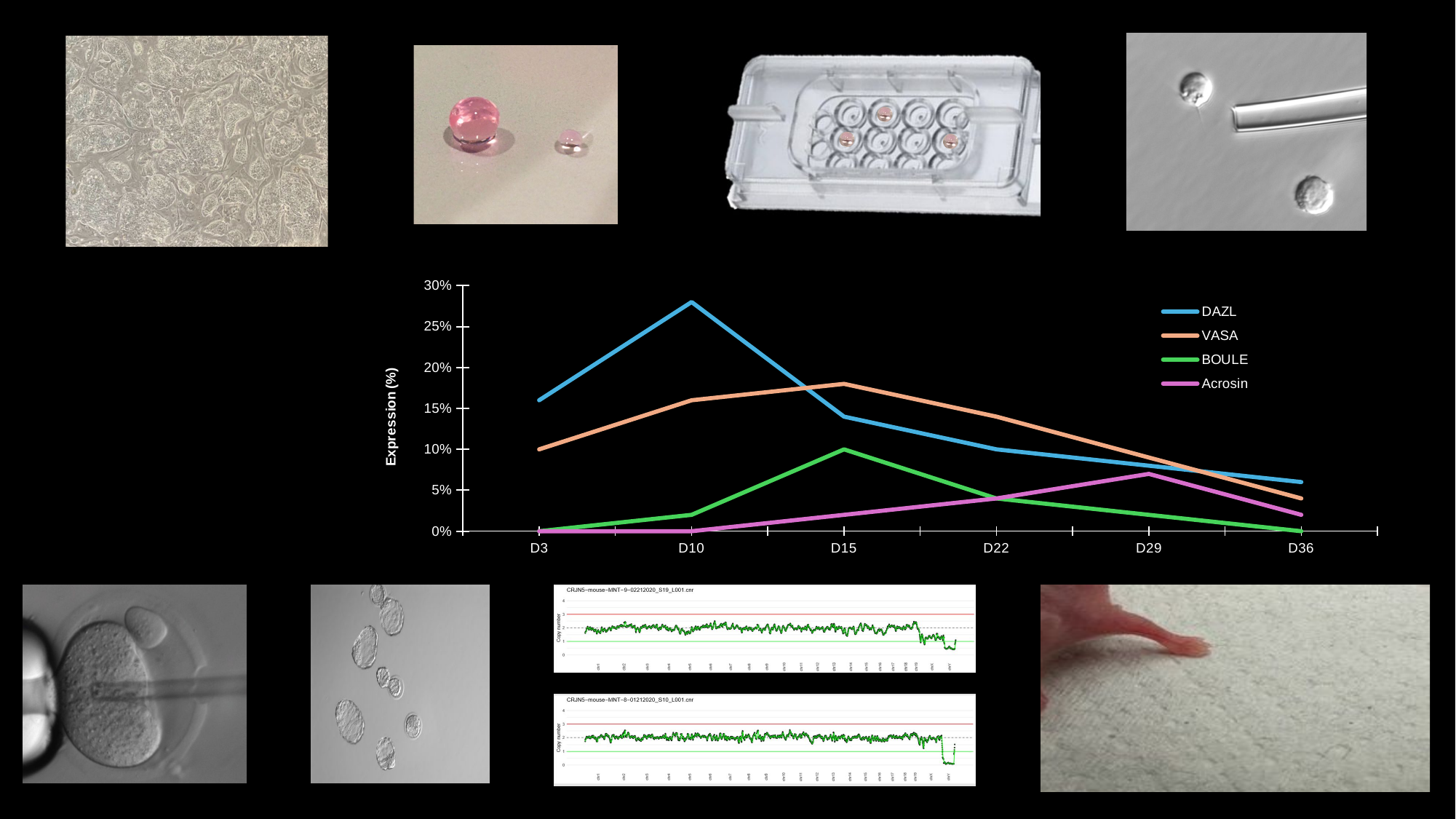

### Chart
| Category | DAZL | VASA | BOULE | Acrosin |
|---|---|---|---|---|
| D3 | 0.16 | 0.1 | 0.0 | 0.0 |
| D10 | 0.28 | 0.16 | 0.02 | 0.0 |
| D15 | 0.14 | 0.18 | 0.1 | 0.02 |
| D22 | 0.1 | 0.14 | 0.04 | 0.04 |
| D29 | 0.08 | 0.09 | 0.02 | 0.07 |
| D36 | 0.06 | 0.04 | 0.0 | 0.02 |

Supplement: gaag036_Supplementary_Data [file gaag036_supplementary_data.zip › Spherification_v1_Graphical Abstract.pptx]
